# Parity of esteem and systems thinking: a theory informed qualitative inductive thematic analysis

**DOI:** 10.1186/s12888-022-04299-y

**Published:** 2022-10-19

**Authors:** Janine Owens, Karina Lovell, Abigail Brown, Penny Bee

**Affiliations:** National Institute for Health and Care Research Applied Research Collaboration Greater Manchester (NIHRARC GM), Faculty of Biology, Medicine and Health, The University of Manchester, Oxford Road, Manchester, M13 9PL England

**Keywords:** Parity of esteem, Systems thinking, Qualitative

## Abstract

**Background:**

Parity of Esteem (PoE) is about equality between mental and physical health but is a term lacking definition and clarity. The complexity of the field of mental health and the conversations around PoE add to its opacity. Therefore, the aim of this study is to use systems thinking to explore the strengths and challenges of using PoE.

**Methods:**

This is a secondary analysis of descriptive qualitative data, from 27 qualitative interviews, utilising the World Health Organisation (WHO) system domains as a framework for the inductive thematic analysis.

**Results:**

Examining the current strengths and challenges of systems in mental and physical healthcare using the WHO domains and macro, meso and micro levels, identifies specific actions to redress inequity between mental and physical health provision.

**Conclusion:**

The evidence suggests that moving PoE from rhetoric towards reality requires new configurations with a systems orientation, which uses macro, meso and micro levels to analyse and understand the complexity of relations within and between domain levels and reorienting funding, training and measurement. This requires embedding new competencies, infrastructures and practices within an effective learning healthcare system.

## Introduction

There are large inequalities between the physical health of people with and without mental health conditions. Inequalities manifest as a significantly higher risk of dying earlier from preventable physical illnesses among those diagnosed with mental health difficulties and pervasive barriers to accessing healthcare [1–7].

Parity of Esteem (PoE) is the idea that mental health receives as much priority as physical health in order to reduce health inequalities [2]. However, it is a term lacking in clarity and viewed as challenging and fraught with bureaucratic, regulatory and structural complexity [8, 9]. In many instances, PoE is merely rhetorical and misleading because it detracts attention from more important questions [10, 11]. There remains opacity regarding the definition of Parity of Esteem and its use in health and social care settings.

All healthcare systems are complex and arguably the field of mental health is even more complex [12], with ‘contested ideas, shared responsibilities and limited evidence to guide interventions and service improvements’ [13]. Moreover, the physical care of people with mental health conditions is complex because disparate teams of health care professionals in different locations deliver these two elements of patient care, this separation reduces continuity of care and inhibits recovery [14].

One way of reframing the numerous conversations and complexity surrounding PoE is to employ systems thinking. This aims at looking how things connect together in order to make up a whole, instead of breaking them down into their constituent parts [15].

Systems thinking is useful in explaining how complex systems work, for example, Bishai et al. used systems thinking by employing a dynamics resource model to explain the political influences on health allocation spending in the public sector and its unintended impacts [16]. Bishai et al. use a system dynamic, one system methodology, whereas other researchers may use other types of systems thinking based approaches. For example, soft system methodology (SSM), which is an action-oriented approach, where a situation is explored using a set of models focusing on purposeful action to inform general problem solving and ways of managing organisational change [17]. Then critical system heuristics (CSH) provide a reference system, limited by four sources of influence, namely; values and motivations, power structures, the knowledge basis and the moral basis [18]. Collectively, these sources of influence provide a richer picture of a situation, forming the basis for developing a more refined systems model, often used in new public health because it fits well with the ecological model [19]. Alternatively, there is the Viable System Model (VSM), developed by Anthony Stafford Beer [20, 21], which sets out the conditions needed for a system to be viable and has previously been used for quality improvement in healthcare [22].

Systems thinking can therefore highlight where research can provide more evidence to guide interventions and improvements. The utility of a systems-level approach may overcome at least two shortcomings in current PoE implementation: i) the lack of specificity in its operational components and progress indicators, making it difficult to define and measure [23] and ii), the sub-optimal ‘siloed’ approach derived from the rigid hierarchical organisational matrices in healthcare and the lack of understanding and co-operation between professional groups with different expectations and pathways [24–26], preventing its redress.

The World Health Organisation [WHO] views systems thinking as an approach to problem solving where problems are part of a wider dynamic system demanding deeper understandings of the linkages, interactions and relationships between elements characterising the system as a whole [27]. The WHO framework outlines the core building blocks necessary for developing and sustaining effective health systems. The six critical domains are; financing- ensuring health financing is adequate and ensures people can access and utilise services without additional costs; service delivery- ensuring, effective, quality and safe interventions are available to those in need with minimum waste on resources; the health workforce-ensuring there are adequate numbers to deliver responsive, fair and efficient services; information- ensuring the production, analysis and dissemination of timely, and reliable evidence on health status, systems performance and determinants of health; medicines and technology- ensuring medical technologies, vaccines and other technologies are scientifically sound, effective, safe, cost-effective and quality assured; and leadership and governance- ensuring strategic policy combines with system design, accountability, regulatory frameworks, incentives, coalition building and effective oversight [27].

In theory, improvements to the quality, responsiveness and coverage of these six domains should lead to improved health equity and parity. However, systems thinking enables a shift in thinking from a more linear and segmented view towards more complex systems founded on synergistic components occurring at macro, meso and micro levels [27–29]. These different analytical levels are important because legislation, policy guidance and financing decisions occur at the macro level, which influences commissioning and implementation at the meso or service delivery level and exerts an impact on how services are received and interactions occur at the micro or individual level. Research can occur at any one of these levels or at all three. However, using systems thinking implies using all three analytical levels because it aims to look at a system as a whole.

This paper is a secondary analysis of data, which frames PoE within a systems-based approach, at macro, meso and micro levels, aiming to examine the current strengths, and challenges of this concept in mental healthcare and attempting identify specific actions to redress inequity between mental and physical health provision.

## Methods

This is a secondary analysis of existing data from a study exploring equality between physical and mental health. The question for the current study is ‘In what ways does system thinking aid in identifying actions to address inequity between physical and mental health?’.

The study conforms to the consolidated criteria for reporting qualitative research [COREQ] guidelines, using a descriptive qualitative design, informed by the WHO health systems framework [30].

### Sample and recruitment

Study participants were local, regional or national key informants working in mental health policy and practice (*n* = 27). Purposive, snowball and theoretical sampling identified local and national key informants. The basis of selection rested on the premise that participants possessed a critical understanding of parity of esteem within and across sectors.

Twenty-seven participants self-selected by agreeing to an interview and providing their contact details. Participants included mental health care providers, physical health care providers, policy-makers, service commissioners, mental health charity workers and political party members (see Table 1). Researchers promised participants anonymity and because of ethical considerations, no further descriptions of characteristics of the sample occur.

**Table 1:** Characteristics of participants

**Role**	**N /27**
Mental healthcare providers	10
Physical healthcare providers	9
Service Commissioners	1
Mental Health Charities	1
Politicians	2
Public Health Professionals	2
Medical education providers	2

### Ethical process and consent to participate

The University of Manchester proportionate ethical review committee gave favourable review for the original study [Ref. no. 2020–8567-15631]. Participants gave informed consent and consented to the use and analysis or re-analysis of data for publication and teaching purposes. This is a secondary analysis of the data and as such conforms to participants consenting to the use of their data. The datasets analysed during the current study are not publicly available because they contain identifiable data. The study conforms to the ethical norms and standards in the Declaration of Helsinki.

### Analysis

This was a secondary analysis of the data from the original study and focuses on redressing inequity between physical and mental health provision, using the domains from the WHO systems framework [27] as a lens through which to view the data and develop themes. It also applied macro (policy), meso (organisational) and micro (individual) levels to identify barriers and facilitators. Analysis was inductive and thematic [31–33] which produced themes mapping onto the six WHO system domains.

Table 2 illustrates the various themes mapping onto system domains and levels.

**Table 2:** Themes mapping onto domains illustrating barriers and enablers at macro, meso and micro levels

**WHO Systems Domain**	**Mapping of themes**	**Explanation of themes and domains**	**Macro Barriers**	**Macro Enablers**	**Meso Barriers**	**Meso Enablers**	**Micro Barriers**	**Micro Enablers**
			**Macro Level**	**Macro Level**	**Meso Level**	**Meso Level**	**Micro Level**	**Micro Level**
Leadership and Governance	• Policy and action plans on parity • Shared decision-making	Working from National level policy to service level delivery to ensure inclusive care, addressing individual need and including families where possible, whilst incorporating parity.	Policy on PoE. Equality Legislation and Standards.			Equivalent policy priority.		
					Lacking insight into patient and carer’s experiences of Mental Health			Sharing decisions with patients and carers or families where possible
Financing	• Funding for services • Targets and incentives • Mental health estate	The impact of inequitable funding for mental health care and inadequate provision, which create an impact on both service providers and users. Targets and incentives should develop holistic care, but limited by their definitions.	Inequitable funding for mental health. Little consideration concerning the complexity of mental health conditions.			Campaigning for funding.		
				Inadequate resource allocation.Different funding models.Lack of investment in the mental health estate.Unclear targets.Targets fail to consider people with complex mental and physical health problems.				
Service Delivery	• Access to quality care • Integrated care and collaboration • Informal caregiver involvement • Person-centred care	Accessibility and availability of services including issues such as acceptable waiting times, medication reviews, follow-ups and availability of a range of services. Integrated multidisciplinary teams learning from one another and working together with families of individuals to provide continuity of and person-centred pro-active care that takes into account differences in social environment and levels of available support				Equivalent clinical priority		
				Inaccessible and ‘patchy’ services.Poor consistency of service provision.Variable availability between areas and regions.Inadequate crisis care.Lack of placed based care.Long waiting lists for children’s mental health.Poor integration of teams.			Suggested multiple access points to gain entry to servicesIntegrating care into the community	
					Lack of time.			Involving families, formal carers and individuals in the decision-making environmentPerson-centred care
Workforce	• Education, training and continuous professional development (CPD) • Staffing numbers	Education and training for all healthcare workers to include mental health, alongside CPD for understanding the aims of the Mental Capacity Act and reinforcing insight into difference. Ensuring adequate staffing of services to ensure equitable delivery of care	Agreement of educational curricula without consideration of meso and micro levels.					
				Poor staffing levels.Workloads.Lack of focused education, training and CPD for staff enabling consideration of the holistic body.Lack of integrated mental health awareness.			Having an understanding of the impact of the social determinants of health on patients and carers	
					Discriminatory staff attitudes.Lack of cultural competence.Inability to engage with diversity.			
Information and Research	• Access to reliable data • Measurement and bench-marking • Parity in research	Access to reliable data in order to improve systems. Measurement and benchmarking for conditions to provide indicators that are more reliable. Parity in research funding to explore and improve outcomes	Lack of parity between physical and mental health research. Lack of reliable data. Lack of clear indicators. Measurement uncertainty for indicators. Ambiguous benchmarking. Introducing and then removing QOFs.			Equivalent research priorities for physical and mental health. Providing Quality Outcome Frameworks (QOFs).		
Technologies and Medical Products	• Choice of treatments • Shared information systems	Access to and choice of ensures equitable access to the latest evidence based treatment and products. There are also shared information systems to ensure joined-up care. Electronic records are linked and available nationally at varying levels to different professionals and primary care systems		Lack of accessibility for electronic records at all levels.				
					Patients prevented from making choices because of perceptions about their mental health status.			Patients and carers enabled to make decisions and choices about treatment.

Table 3 illustrates the stages of inductive thematic analysis and the ways trustworthiness was established.

**Table 3:** Stages of analysis and establishing trustworthiness

**Stages of Thematic Analysis**	**Establishing Trustworthiness**
Stage 1: Data Familiarisation	JO immerses in data and researcher field notes, documents reflections, initial thoughts about codes and themes
Stage 2: Initial generation of codes	JO feeds back initial thoughts to AB, PB, KL for comments, defines coding framework, documents team meetings and decisions
Stage 3: Initial Theme identification	JO triangulates data from theoretical sample, carries out researcher triangulation with AB, makes a diagrammatic map of theme connections and notes about how decisions were made
Stage 4: Reviewing themes	Themes and subthemes reviewed by AB, KL & PB. JO returns to raw data to review and consider whether anything has been omitted and if interpretation ‘fits’
Stage 5: Defining and naming themes	JO returns to field interviewer AB to triangulate data and considers theoretical triangulation with existing published evidence for confirmability. JO, AB, KL& PB discuss themes and reach consensus.
Stage 6: Producing the paper	JO feeds back findings informally to participants wishing to remain involved (carries out member checking), explains reasoning for choices and the processes incurred. JO drafts the paper and this is then fed back on by KL, AB & PB.

### Triangulation of data

Different forms of triangulation increased trustworthiness; the study used investigator triangulation through different researchers for interviewing (AB and VB) and analysing the data (JO, AB, KL, PB)- see Table 3 for further detail. Norman Denzin argues examining research occurs from multiple perspectives including those of ‘multiple observers, theories, methods, and data sources,’ with the intent of overcoming the ‘intrinsic bias that comes from single-method, single-observer and single-theory studies’ (p. 307) [34]. JO used theoretical triangulation whereby different theories were compared to participants’ own accounts with what Denzin calls ‘alternative theoretical schemes’ (p. 1023) [34] because it employs multiple, rather than single perspectives to explore an area. This enabled differences, similarities and inconsistencies in the data to emerge [35] and build a plausible account.

### Reflexivity

Reflexivity is the gold standard for ensuring trustworthiness in research [36]. AB was of a different ethnicity, reflecting diversity in the sample. The age of AB reflects the younger ages of the sample and the ages of JO, KL & PB reflects the older ages. AB is a junior researcher and JO, KL & PB are senior researchers, reflecting the education and experience of the sample. KL is a clinical academic, PB is an applied academic researcher and JO is a social scientist from a different cultural background who focuses on critical approaches within qualitative research. These different backgrounds and identities produced multiple perspectives during the process of analysis.

## Results

The results present the six WHO domains: leadership and governance, finance, service delivery, workforce, information and governance and themes mapping onto the domains, alongside barriers and enablers of PoE at macro, meso and micro levels.

### Leadership and governance

Key themes for this domain included a) policy and action plans on parity and b) shared decision-making. Policy and action plans at the macro level for parity of esteem appear to have created barriers due to a lack of clarity and definition in the ways that parity may be achieved [37–39]. Broad enablers for PoE at the macro level included “campaigning for mental health funding” (PT 19), and “making sure mental health problems have equivalent policy priority” (PT 18), but the method of enactment of these initiatives required meso and micro level thinking, there appeared to be no meso level thinking for policy and action plans and this may be an area worthy of further research to produce more evidence. Participants argued that setting equality standards at the macro level such as everyone having a maximum of a two-week wait prevented shared decision-making, creating barriers because it worked against people experiencing a mental health crisis, tending to treat all mental health conditions as the same with little consideration for diversity, or complexity. “Why is it that people with psychosis, or a psychotic crisis, should expect the equivalent, or parity, if you like, of access, as someone with […] cancer?”(PT1).

Shared decision-making inevitably links to patient-centred care, and equity in this context meant having the autonomy to build a relationship and respond to a patient as a person situated in a particular psychosocial and economic environment. The majority of this activity occurred at the meso and micro level. “To understand that what generates good diabetic care, is often understanding psychological and social contexts in which people have their difficulties” (PT1). However, there were inconsistencies in responses from participants with some exhibiting a lack of insight into patient and carer experiences of mental health, which had an impact on the positive practice of including patients and families in decision-making.

### Financing

Key themes for this domain included a) funding for services, b) targets and incentives and c) the mental health estate.

All participants mentioned the central role of funding; suggesting that a system was constrained by inequitable funding was both a challenge to and a manifestation of a lack of PoE. Participants perceived that moving funding around to prioritise one area over another perpetuated mental and physical health inequalities, inadvertently setting the two fields up as financial competitors rather than an integrated healthcare goal. “In a resource constrained system, if we want to give more to someone else we have to take it from one area and move it to another” (PT 27). There were no enablers at the meso and micro level and no barriers or enablers mentioned at the micro level leaving a gap in the current evidence.

Integrated care systems occur at meso levels but remain heavily dependent on funding from the macro level, with mental health appearing secondary to physical health “there's always going to be people with what appear to be hugely significant health problems physically that “trump” mental health problems” (PT 27). Participants mentioned further local tensions in resource allocation and funding for acute and mental health services, emanating from this inequity. “[…] each ICS (integrated care system) has a sort of capital spending limit, which has to be shared between its acute hospitals and its mental health trusts. Mental health is always a relatively small player” (PT 6)*.* Participants believed that this Cartesian vision of mental and physical health limited the ability to gain larger amounts of funding from the macro level.

Participants also discussed the inadequacy of mental health funding for children and adolescents, suggesting local and whole system inequalities maybe further exacerbated in some populations. “What we focus on (young people and mental health)* –* is so sort of underfunded and under-prioritised compared to the rest of the healthcare system” (PT 12). Interrogating this over-generalization further in future could explore the factors that lead to and ways of reducing inequalities using a whole system approach.

Participants perceived a need for adequate resourcing at meso levels to meet policy and service targets “the need is to focus resources and provision to meet those targets. I think the challenge with it is that you have to have adequate resources to do that” (PT 21). However, targets added a new level of complexity that risked whole system improvement. “The danger with that is we get narrowly focused on how many people are accessing psychological therapies, and a more encompassing measure looks at people who maybe don’t want to access psychological therapies, but do still want support” (PT 2). Targets did not appear to address provision for people with complex mental and physical health problems, resulting in a lack of parity. “There are waiting time targets for psychological therapies, but it seems that not really that much is done around those people who fall through the gaps and who are too complex” (PT 2). The complexity of conditions appeared to be an area receiving very little consideration at macro level when allocating funding. “The other big test of the complexity of parity of esteem, in a sense, is that people don't have one problem. So people don't just have a psychological difficulty, or they don't just have diabetes, they often have multiple problems” (PT 1). At worst, participants suggested that targets might lead to a reduction of total effort at the meso level “sometimes what happens then is people only focus on the targets, rather than the bigger picture” (PT 5).

A considerable challenge involves clearly identifying indicators for benchmarking PoE progress “the indicators are going to act as this kind of galvanising force around an area that the targets are going to do that so you have to be very specific about what it is you want change” (PT 8).

Participants perceived that a lack of funding at the macro level affected both the physical environment of mental health services at meso level and the availability and quality of care for people with mental health problems at micro level. A lack of investment in physical infrastructure affected mental health care creating “facilities that are not fit for purpose, with people still in dormitories and wards that are not acceptable in this day and age” (PT 6). A concomitant impact on patient wellbeing and long-term recovery was evident. “Often in emergency departments there will be a particular area or a particular room where someone who is experiencing a mental health crisis, or presenting with a primary mental health need, will be placed. Often those places aren’t very well looked after; they’re not very welcoming, they’re not very therapeutic.” (PT 25). What may assist here is developing more insight at policy level on the complexity of conditions and the ways of incorporating this to create more flexibility and effective guidance.

### Service delivery

Key themes in the service delivery domain were a) Access to quality care, b) Integrated care and collaboration, c) Informal caregiver involvement and d) Person-centred care.

Macro level enablers for participants included “making sure mental health problems have equivalent clinical priority” (PT 18).

Crisis services in particular exhibited many barriers to support and care at a meso level. “How easy it is for people with mental health problems and their families to get support when somebody’s in crisis. How responsive is that service? The answer is it isn’t” (PT 11).

Participants discussed the accessibility of formal services and the sub-optimal role that GPs may play as gatekeepers to this system “It’s no criticism of GPs, but Access Points rely heavily on people going through GPs to access the system.” (PT 3). Potential solutions such as meso level enablers involved multiple access points and better health care integration and support within communities. “There needs to be equity of access once you pass the threshold and therein lies the problem, because access for someone with a mental health problem, even to a service which has parity of resource, has to be different to someone with a physical health problem.” (PT 27).

Participant’s perceptions also focused on the importance of availability of services at meso level “The early intervention psychosis programmes are really good examples. There aren't enough of them and people can't get at them and, you know, so they're patchy, it's not uniform.” (PT 25). Barriers to early intervention services and crisis care came up numerous times in people’s narratives across adult, child and adolescent services. Given the possible impact of early mental health difficulties across the lifecourse, participants viewed deficits in children’s care as particularly salient. “If you have a child who is anxious and depressed, to the point it's getting in the way of their development, and they are ten years old, being on a waiting list for a year, is a tenth of their lifespan that they’ve not been functioning for and they pay a very heavy developmental price.” (PT 5).

A lack of continual and follow-up support for people and their supporters when experiencing mental health crisis reinforced a lack of parity for acute and mental health service user, with caregivers seen as instrumental in negotiating access and responsiveness from mental health services. Perceived enablers at the meso level involved carers and families; “Family members are a really important part of navigating the health systems, advocating for the health systems, reminding the health systems, they alert if there's a problem going on. […].” (PT 1).

Participants discussed involving families and formal carers as facilitators for developing greater levels of acceptability. Without the support of families and formal caregivers, professionals may struggle to deliver acceptable, effective and equitable care, but this itself raises challenges at the micro level in terms of language used, clinical culture and power differentials.

Barriers to patient-centred care involved time and pressure on services “time is an important factor because of increasing service demands and pressure on clinical services. Sadly, even with the best will, we don’t have the time to sometimes delve into a little more detail.” (PT. 24). Without the time to explore with patients and their carers important details and inclusion in the patient professional encounter then ‘no decision about me without me’ [40] (p.3) is unlikely to occur. Some participants discussed person-centred care as both a facilitator and barrier to parity. “Making sure that the patient is able to tell you more about their needs than anything else” (PT16). However, “With mental health I think everyone is a bit more fearful; they remove themselves, they maybe don’t want to enter in a discussion” (PT 23). It would appear that for parity to occur in choice and decision-making there needs to be more training in facilitating individuals, families and carers to become part of the decision-making environment.

### Workforce

Key themes for this domain were a) Education, training and continuous professional development (CPD), and b) Staffing numbers.

Participants perceived the impact of lack of funding on staffing numbers at macro level as a challenge to service delivery at the meso level “When patients come into ED, they’re often waiting for the mental health liaison team. They’re quite often short-staffed, underfunded” (PT 23). They felt that a lack of resources had a direct impact on staff themselves, particularly in early intervention and crisis care services, which then exerted an effect at the micro level: “the staff are under so much pressure, they have no resources and not enough staff” (PT 22).

“When someone’s in a mental health crisis, they should not be left to not know where to go. Or to not have support because there aren’t enough resources or the service isn’t open” (PT 2).

Staffing was an issue throughout participant interviews, particularly at the meso level for the workforce is a substantial resource within the services, and arguably, it is important to keep abreast of new evidence and ensure holistic care is prominent. Some participants highlighted a siloing of education and training which focused mostly on physical health “If I'm trained as a physician I'm trained in physical health. I learn to be blind to mental health problems” (PT 27). Cartesian duality appeared reified by education “you know we’re definitely trained in splitting people's bodies and minds.” (PT 1). This disjointed training at the meso level disabled healthcare practitioners, affecting service delivery at the micro level, because there are barriers to effective communication and asking people about their mental health.

### Information and research

Key themes for this domain were; a) Access to reliable data; b) Measurement and benchmarking; and c) Parity in research.

Acknowledgement came from participants regarding the lack of parity between physical and mental health research “you’d want to see some parity in mental health research. Of course, it’s way behind other health areas.” (PT 14). Reducing barriers at the macro level would facilitate more equitable meso level advances. Whilst broad macro level enablers for participants included “making sure mental health problems have equivalent research and funding priority” (PT 18), more specificity was evident in some participant’s responses.

Participants discussed meso level barriers to accessing reliable data because of variations in coding and collecting data. “Quality of the data is still really poor and the way that different NHS trusts collect the data, there’s just a huge amount of variation. How can you assess the effectiveness of the system, the quality of care, if first of all you’re not collecting the right data?” (PT 12). Enablers involved standardising data aiming towards quality improvement and “really understanding the data and what you’re measuring, really understanding the change cycle that you’re implementing, and then sustainability about the metrics that you’re collecting, to measure it.” (PT 2). Another participant suggested that there was a pressing need “to invest more time and energy and effort and research into what patient reported outcomes (PROMs) would be of value to people.” (PT 24).

Being able to judge the efficacy of a system and need for resources becomes challenging if the data is unreliable. It also makes requests for further resources difficult.

Within healthcare, benchmarking links to targets, incentives and metrics and usually classified into one of four categories: productivity, quality, time and cost-related. This allegedly establishes standards of excellence and improves services and quality [41]. Participants perceived that benchmarking was important “how are we doing on this indicator, compared to the national average? I would do it more by bench-marking the key indicators.” (PT 14). This also underlined considerable operational complexity: “How you measure what the outcomes are for something like mortality? Do you then break it down for different mental health conditions?” (PT 3).

At the meso level, participants gave positive examples of enablers such as Quality Outcomes Frameworks (QOFs) involving integrated care addressing physical and mental health. “They access a dietician, get to the optician, see the podiatrist, and go for their regular physical health checks. They should have an ECG and bloods taken at regular intervals at least once a year. People with bipolar disorder and schizophrenia should have that in general practice. There was a QoF around that, but that’s been removed” (PT 17). While participants perceived incentivising physical and mental health through the introduction of yearly health checks at the meso level as an enabler, the potential for the mis-coordination or instability of these approaches was a prominent barrier. This links strongly to the funding domain.

### Technologies and medical products

Key themes for this domain were a) Shared information systems and b) Treatment choice.

This domain was least mentioned. Participants who did discuss PoE under this domain typically perceived barriers to care emanating from a lack of shared information systems at the meso level. This occurred primarily when they were attempting to support inpatients with mental health care. Inflexible interpretations of confidentiality and data protection appeared to present specific barriers. For example, blocking access of healthcare staff to scans and appointments sent to the patient’s home address, even though they were in-patients in a mental health unit in the same hospital grounds:

“They have a system within the main hospital where all the CT scans are reported and actually held electronically, but we don’t have access. We have tried to get access and we have failed. The radiologists are telling us, ‘we cannot give you access to the system, we cannot email you the result, and you have to physically come’. So, we have to leave our building, walk across the hospital site, go to another building and get the scan result. When my junior doctor arrived there they said, ‘oh no, we are not giving it to you’. So, they can’t email it to us, they can’t physically give it to us, they can’t put it on the system, so I’m not really sure how we’re supposed to access it.” (PT 17).

Other participants discussed barriers to treatment choices resulting from perceptions around mental health at the micro level “I would always discus treatments and the choice and the availability of different treatments to a patient and, you know, involve, them […]. In mental health, for some reason, a different idea is that they can’t make that decision or they shouldn’t have that decision” (PT 23). This appears to suggest that professional or institutional discrimination might result in patients with mental health problems ultimately having less choice and by implication iniquitous or sub-optimal care standards. This links to leadership and governance, workforce and service delivery domains.

## Discussion

**Fig. 1:**
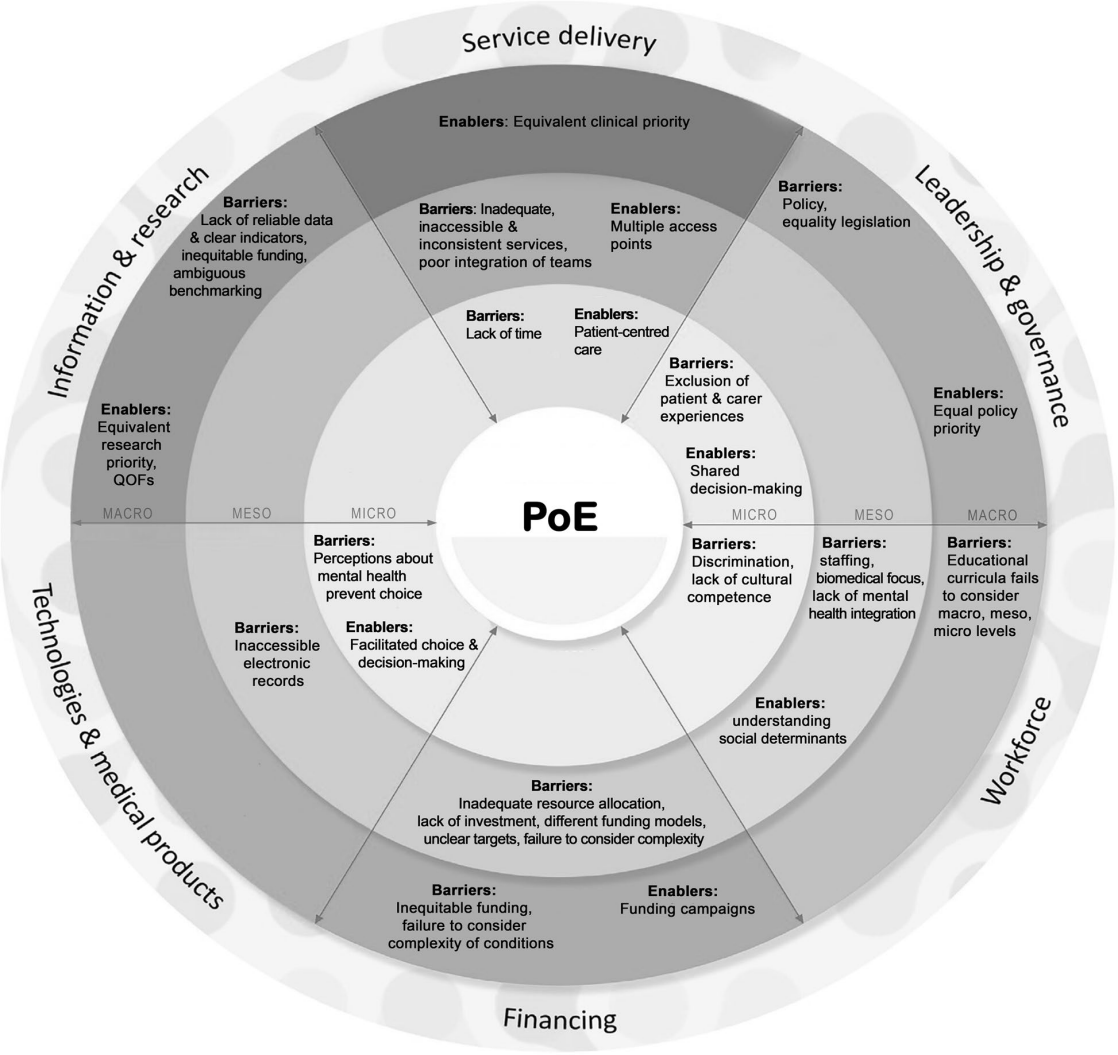
Conceptual model of interactions between domains and levels

The aim of this paper was to frame PoE within a systems approach in order to deconstruct it into clearly defined domains and system levels and describe facilitators and challenges. Delineations between macro, meso and micro levels are not always clear, for example, in the domain of service delivery, training deficiencies are meso level but could also be micro level because this affects patients. Another argument is that it could be macro level because policy decisions can alter healthcare curricula or CPD requirements. Therefore, context is important. Table 2 displaying the domains and levels may not be comprehensive, but for this study, participants identify more barriers at macro and meso and micro levels across domains than enablers for PoE**.** This is difficult to explain because it is only a small-scale qualitative study, employing the perspectives of professional participants and interpretation could differ if the perspectives of service users were included. Figure 1 is a conceptual model of the interactions between the domains.

The domain for Leadership and governance exhibited enablers at macro and micro level through policy and guidance, but simultaneously participants viewed these actions as barriers because of a lack of clarity in definitions, guidance and little acknowledgement of the complexity of physical and mental health conditions. Although, it should exert an influence at meso and micro levels, there was no evidence of leadership and governance at meso level, leaving decisions about PoE to individuals in the service delivery and workforce domains. Setting standards at the macro level such as a two-week wait for all conditions created inequity because of the failure to acknowledge difference and complexity between and within conditions and people. Shared decision-making at the micro level acted as an enabler for parity, but a barrier at the micro level, because of a lack of time, creating a negative impact in the service delivery domain.

Financing exerted an impact and interacts across all domains and levels. Participants suggest that a lack of funding and resources creates inconsistencies in resource allocation, care quality and outcome measurement. The failure to consider complexity remains unaddressed, affecting multiple health system domains and implementation levels. This is an area where further research could provide a template for more flexibility in policy decisions; considering the complexity of conditions. Governments rightly aim to achieve real value-for-money through public investment in service delivery. However, the lack of recognition concerning the complexity of the environment and the impacts of policy changes on service delivery appears to exclude equitable care improvements and measurement. For mental health, outcome measures need to be valid, reliable, sensitive to change, comparable across a range of service users and meaningful for both clinicians, patients and their supporters [42, 43]. The main problem that the participants in this study identify is that implementing outcomes reactively maintains contractual and funding requirements rather than enacting well-defined and appropriate goals, which may more readily lead to equity.

At micro-level, enablers may include the introduction of more meaningful outcome measures reflecting improvement in social and occupational functioning, mental health symptoms and distress, and physical health and wellbeing. This intention would strengthen progress in the information and research system domains, because it interacts with all domains and provides evidence for technologies and medical products, workforce in terms of training and staff allocation, service delivery in terms of care allocation and environment and adequate financing. Taken together, exploring how strengthening of one domain exerts a significant positive impact on other domains could be useful for future research. Whilst outcomes measurement offers valuable information on the impact and effectiveness of service delivery, it also presents its own challenges. For example, valid outcome measurement may be dependent on the availability of reliable evidence and data capture tools and affected by the complexity and interactions of other systems at meso level.

The Service delivery domain interacts with domains for financing, the health workforce, information, medicines and technology and leadership and governance. Instead, it focuses heavily on ‘Access to care’, which demonstrates intrinsic and direct links to outcomes measurement and well-defined goals instigated at the macro and meso levels [44]. Access to quality care is currently difficult to measure with certainty because it is a theoretical concept encompassing different aspects and dependent on the exact definition and the context of measurement [45]. Enablers at the meso level may include multiple access points to support services in the community, extending the reach of community support services and facilitating access to crisis care before symptoms or behaviour becomes unmanageable. These initiatives may present as quantifiable improvements at the micro level through continual review, follow-up with patients and family and caregiver support to prevent escalation of symptoms. Research suggests that including family members in decision-making and choices is a positive approach to mental health, as long as agreed by all [46, 47].

The Workforce domain, interacts with finance, information and research provide evidence for care and use of medical technologies and products, which are implemented through service delivery and guided through leadership and governance. However, the siloing of education and the creation a Cartesian divide appears in urgent need of address, leading from a macro-level reorientation of training, education and CPD focused on the holistic body. This has potential to initiate gains in multiple system domains, such as the interaction with the Technology and Medical Products domain at the micro level where stereotypical perceptions about mental health may still be limiting treatment choices. It also affects patient-centred care by interacting at the micro level in the Service delivery domain. The financing domain, in itself directly interacts with the workforce domain, dictating staffing levels at the meso level and resources, decided through the service delivery domain and the development or otherwise of a supportive workforce culture.

The concept of a learning health system (LHS), in which an organisation builds knowledge or evidence, embeds quality improvement practices as standard practice, actively engages patients and family members and supports further learning [48], offers a potentially fruitful approach to building parity across the multiple interacting domains and levels of healthcare. Taking these four elements of the learning healthcare system and using them in a PoE focused systems-based approach may offer important traction.

### Study limitations

The sample limited collection of data during lockdown in 2020/2021. Original data collection was for a larger study on PoE and this is a secondary analysis of that data. Our assessment of macro, meso, micro levels within the study represents a professional perspective, and future studies may garner a more comprehensive insight from everyone involved across different settings and levels.

## Conclusion

Moving PoE from rhetoric to reality suggests the need for new configurations that have a systems orientation. This includes new approaches from macro to micro levels, which focus on reorienting funding, research, training and measurement, oriented towards understanding the complexity of relationships within and between domains and levels. This requires ongoing development, monitoring, evaluation and may most usefully occur by adopting a whole systems approach and embedding this principle within an effective learning healthcare system.

## Data Availability

The qualitative data generated and analysed during the current study are not publicly available because they contain personal data, for example, job roles, place of work, colleague names, family names and circumstances, which could identify the participant. Redacted data are available from the corresponding author on reasonable request.
